# Enhanced Chondrogenic Differentiation of Human Umbilical Cord Wharton's Jelly Derived Mesenchymal Stem Cells by GSK-3 Inhibitors

**DOI:** 10.1371/journal.pone.0168059

**Published:** 2017-01-06

**Authors:** Prapot Tanthaisong, Sumeth Imsoonthornruksa, Apichart Ngernsoungnern, Piyada Ngernsoungnern, Mariena Ketudat-Cairns, Rangsun Parnpai

**Affiliations:** 1 Embryo Technology and Stem Cell Research Center and School of Biotechnology, Suranaree University of Technology, Nakhon Ratchasima, Thailand; 2 School of Anatomy, Institute of Science, Suranaree University of Technology, Nakhon Ratchasima, Thailand; Louisiana State University, UNITED STATES

## Abstract

Articular cartilage is an avascular, alymphatic, and aneural system with very low regeneration potential because of its limited capacity for self-repair. Mesenchymal stem cells (MSCs) are the preferred choice for cell-based therapies. Glycogen synthase kinase 3 (GSK-3) inhibitors are compounds that can induce the Wnt signaling pathway, which is involved in chondrogenesis and cartilage development. Here, we investigated the influence of lithium chloride (LiCl) and SB216763 synergistically with TGF-β3 on chondrogenic differentiation in human mesenchymal stem cells derived from Wharton’s jelly tissue (hWJ-MSCs). hWJ-MSCs were cultured and chondrogenic differentiation was induced in monolayer and pellet experiments using chondrogenic medium, chondrogenic medium supplemented with LiCl, or SB216763 for 4 weeks. After *in vitro* differentiation, cultured cells were examined for the expression of *Sox9*, *ACAN*, *Col2a1*, and *β-catenin* markers. Glycosaminoglycan (GAG) accumulation was also examined by Alcian blue staining. The results indicated that SB216763 was more effective than LiCl as evidenced by a higher up-regulation of the expression of cartilage-specific markers, including *Sox9*, *ACAN*, *Col2a1* as well as GAG accumulation. Moreover, collagen type II expression was strongly observed in cells cultured in the chondrogenic medium + SB216763 as evidenced by western blot analysis. Both treatments appeared to mediate the Wnt signaling pathway by up-regulating β-catenin gene expression. Further analyses showed that all treatments suppressed the progression of chondrocyte hypertrophy, determined by decreased expression of *Col10a1* and *Runx2*. These results indicate that LiCl and SB216763 are potential candidates for further *in vivo* therapeutic trials and would be of great importance for cartilage regeneration.

## Introduction

Articular cartilage is a highly specialized connective tissue of the synovial joints. Chondrocytes are specialized cells in this tissue responsible for the generation of the extracellular matrix (ECM) and the maintenance of the tissue function. Generally, articular cartilage injuries cannot self-repair due to the lack of vascular, lymphatic, or nervous systems [1].

An alternative approach of cartilage preservation and repair that uses stem cell-based therapies such as mesenchymal stem cells (MSCs) was recently developed. MSCs can be isolated from the bone marrow [2], adipose tissue [3], dental pulp [4], umbilical cord blood [5], and Wharton’s jelly tissue [6]. Several sources of MSCs exhibit different properties of stemness, expansion capacity, and multilineage differentiation [7,8]. Wharton’s jelly tissue (WJ) is an alternative source of MSCs, which show properties similar to MSCs from other sources. It is a rich source of primitive cells [6,9]. In addition, WJ-MSCs have greater proliferation rates, expansion potential, and differentiation potential than other adult MSCs [10]. Thus, WJ-MSCs have been considered a source of candidate cells and present therapeutic potential for cartilage regeneration.

Members of the transforming growth factor-beta (TGF-β) superfamily are the most crucial inducers of chondrogenic differentiation in MSCs such as transforming growth factor-beta (TGF-β) and bone morphogenetic proteins (BMPs) [11]. The Wnt signaling pathway is also involved in chondrogenesis and cartilage development [12]. Canonical Wnt signaling is mediated by β-catenin, which accumulates in the cytoplasm and then translocates to the nucleus. β-catenin forms a complex with DNA-binding T-cell factors (TCFs) to activate the transcription of target genes. The β-catenin signaling pathway often crosstalks with other signaling pathways to modulate chondrogenesis [13–18]. However, the Wnt/β-catenin signaling pathway plays a crucial role in the hypertrophic maturation of chondrocytes during the endochondral ossification process [19]. Another key regulator of the Wnt signaling pathway is glycogen synthase kinase 3 (GSK-3) that mediates β-catenin phosphorylation [20]. GSK-3 inhibition promotes the accumulation of β-catenin and complex with co-transcription factors, LEFs/TCFs, to promote transcription [21,22]. Lithium chloride (LiCl) or SB216763 has the potential to be inhibitor of GSK-3, which inactivates the phosphorylation of β-catenin to initiates the Wnt signaling pathway [23,24]. LiCl was the first GSK-3 inhibitor to be discovered and has been used in the treatment of bipolar disorder [23]. SB216763 is a synthetic small molecule that highly ATP competitive to inhibit the GSK-3 [24].

In this study, we investigated the influence of LiCl and SB216763, which act synergistically with TGF-β3 on chondrogenic differentiation in hWJ-MSCs by monolayer and pellet cultures experiments. The differentiated cells were characterized by GAG analysis, immunofluorescent staining, western blot, and gene expressions analysis.

## Materials and Methods

### Chemicals and reagents

All chemicals and reagents were purchased from Sigma-Aldrich Corporation (St. Louis, MO, USA), unless otherwise indicated.

### Human articular cartilage preparation

This study was approved by the Ethics Committee for Researches Involving Human Subjects, Suranaree University of Technology. Human articular cartilage (n = 1) was obtained from a patient undergoing total knee replacement for osteoarthritis at the Suranaree University of Technology Hospital, Nakhon Ratchasima, Thailand after the patient had signed the informed consent. Human articular cartilage was used as a control in the experiments.

### Isolation and expansion of hWJ-MSCs

The human umbilical cord (n = 1) was collected from Suranaree University of Technology Hospital, Nakhon Ratchasima after patient’s informed consent was obtained. MSCs were isolated from the umbilical cord using a tissue explant procedure as previously described [25]. Briefly, the umbilical cord was washed in sterile PBS and cut lengthwise to open the gelatinous (Wharton’s jelly; WJ) tissue. The vessels were excised and diced into small fragments (about 3 × 3 mm). Then, WJ tissues were plated onto 6-well tissue culture plate (SPL life sciences, Gyeonggi-do, Korea) and then carefully covered with 1 mL of culture medium comprised of alpha modification of Eagle’s medium (α-MEM) supplemented with 100 units/mL penicillin, 100 μg/mL streptomycin and 10% of fetal bovine serum (FBS, Life Technologies Inc. Gibco-BRL Division, Grand Island, NY, USA). Culture cells were then incubated at 37°C in a humidified atmosphere of 5% CO_2_ in air for 7–10 days. Medium was replaced every 2 days and, when visible fibroblast-like cells were observed, then tissue explants were removed. The cells were expanded until passage 3 (P3), then the cells were either directly used for experiments or cryopreserved with 10% dimethyl sulfoxide (DMSO, Calbiochem, San Diego, CA, USA) and stored in liquid nitrogen.

### Immunophenotyping

hWJ-MSCs at passage 5 were cultured onto 4-well tissue culture dishes (Nunc, Roskilde, Denmark) in the growth medium until reaching 70% confluence. Cells were fixed by 4% paraformaldehyde (PFA) for 30 min. Nonspecific binding was blocked by 10% normal goat serum. Primary antibodies were raise against CD34 (BD biosciences, San Jose, CA, USA), CD73 (Millipore, Massachusetts, USA), CD90 (Santa Cruz Biotechnology, Dallas, TX, USA), and CD105 (Santa Cruz Biotechnology) at 4°C overnight. Cells were incubated with secondary antibodies, Alexa fluor^®^ 488 goat anti-mouse IgG (Invitrogen, Carlsbad, CA, USA) or Alexa fluor^®^ 488 goat anti-rabbit IgG (Invitrogen). Nuclei were stained with 4, 6-diamino-2-phenylindole (DAPI, Millipore) and observed under a fluorescence microscope (Nikon Eclipse Ti-S, Japan).

### Multipotency assays

hWJ-MSCs were cultured at the final density of approximately 2 × 10^4^ cells/cm^2^ in 6-well culture plates coated with 0.1% gelatin.

hWJ-MSCs were induced to osteogenic differentiation by culture in the culture medium with reduced FBS to 5% and supplemented with 100 nM dexamethasone, 0.2 mM L-ascorbate-2-phosphate, and 10 mM β-glycerophosphate. The medium was subsequently replaced every 2 days for 3 weeks. Calcium deposits from the cells were then visualized by Alizarin Red staining.

To induce adipogenic differentiation, hWJ-MSCs were cultured in the culture medium with reduced FBS to 5% and supplemented with 10 μg/mL insulin, 100 μM indomethacin, 1 μM dexamethasone, 0.5 mM isobutylmethylxanthine (IBMX). IBMX was removed from this medium after 1 week of culture. The medium was subsequently replaced every 2 days for 3 weeks. Cells were then stained with Oil Red O to observe oil droplets.

To induce chondrogenic differentiation, hWJ-MSCs were cultured in a completed chondrogenic medium consisting of culture medium with reduced FBS to 2% and supplemented with 1% Insulin-Transferrin-Selenium-Ethanolamine (ITS-X, Invitrogen), 50 μg/mL ascorbate-2-posphate, 40 μg/mL L-proline, 100 μg/mL sodium pyruvate, 100 nM dexamethasone, and 10 ng/mL of TGF-β3 (Prospec, East Brunswick, NJ, USA). The medium was replaced every 2 days for 3 weeks. GAG production was assessed by Alcian blue 8× staining.

### Cytotoxicity test

One thousand hWJ-MSCs were re-plated and cultured in 96-well culture plates (SPL life sciences) in the culture medium for 6 h to allow attachment. Next, LiCl or SB216763 cytotoxicity was assessed by adding either LiCl or SB216763 to the culture medium at concentrations of 0, 5, 10, and 20 mM or 0, 1, 2.5, and 5 μM, respectively. All cultures were maintained for 72 h at 37°C in a humidified atmosphere of 5% CO_2_ in air. The effects of LiCl and SB216763 on cell viability were quantified by the MTT assay. Briefly, culture medium was replaced by 5 mg/mL MTT solution (Invitrogen) in culture medium and cells were incubated for 2 h. DMSO (Calbiochem) was then added and incubated at 37°C for 10 min. The absorbance was measured at 540 nm. (Microplate reader Sunrise, TECAN, Austria)

### Chemical induction of chondrogenic differentiation

For monolayer cultures, hWJ-MSCs were cultured and chondrogenic differentiation was induced as mentioned previously. The experiments were performed by dividing the cells into 4 groups. Each group of cells was cultured in culture medium with reduced FBS to 2% (Control), chondrogenic medium, chondrogenic medium supplemented with 5 mM LiCl or 1 μM SB216763. For pellet cultures, 2.5 × 10^5^ hWJ-MSCs were centrifuged at 3000 rpm for 5 min in a 15-mL conical tube (Corning, Corning, NY, USA) to form pellets [24] and then incubated at 37°C in a humidified atmosphere of 5% CO_2_ in air. After 24 h, the pellets were cultured in 4 different media as mentioned above. The tubes were incubated with loosened cap at 37°C in a humidified atmosphere of 5% CO_2_ in air. The medium was replaced every 3 days.

### GAG analysis

Cell monolayer and pellets from the differentiation experiments were fixed with 4% PFA for 30 min at room temperature. Cell pellets were embedded in Cryostat embedding medium (Pink, Killik, Italy) and frozen on dry ice. The embedded samples were cut at 10-μm thickness with a cryostat microtome (CM2850, Hestion, Australia) and placed in the center of a coated slide. Monolayer expanded cells and pellet section samples were examined for GAG accumulation by staining with Alcian blue 8× for 30 min.

### Immunofluorescence staining

Monolayer expanded cells and pellet sections were blocked and permeabilized for 1 h at 37°C with 5% bovine serum albumin (BSA), 5% normal goat serum, and 0.1% triton-X100. For collagen type II and X staining, samples were predigested with 1 mg/mL pepsin and 0.2% hyaluronidase, respectively. Anti-human type II collagen (clone 6B3, Chemicon) and anti-human type X collagen (Calbiochem) primary antibodies were incubated overnight. Samples were incubated for 2 h with the respective secondary antibodies. Then, samples were stained with DAPI and observed under a fluorescence microscope.

### Gene expression analysis

After 28 days of induction, total RNA was isolated from the cells by total RNA extraction kit (RBC Real Genomics, RBC Bioscience, Taipei, Taiwan) according to the manufacturer’s instructions. Then, RNA was reverse-transcribed in the presence of oligo-dT primer for complementary DNA (cDNA) synthesis by iScript^™^ Reverse Transcription Supermix for RT-qPCR (BioRad, Hercules, CA, USA). The expression of several genes was assessed by using Light Cycler^®^ 480 (Roche Diagnostics, Basel, Switzerland) and KAPA SYBR-Green PCR Master mix (Applied Biosystems, Carlsbad, CA, USA). The primers used are shown in Table 1. Melting curve analysis was undertaken to determine the specificity of the primers. Gene expression was normalized to the reference gene *GAPDH* and calculated as the relative expression compared to control cells. The qPCR analyses were performed three times.

**Table 1:** Primers used for qPCR analysis.

Gene	Primer Sequences (5’→3’)	Annealing Temperature	Product Size (bp)	References
*ACAN*	F:ACTTCCGCTGGTCAGATGGA	63	111	[26]
	R:TCTCGTGCCAGATCATCACC			
*Sox9*	F:GGCAAGCTCTGGAGACTTCTG	59	207	[26]
	R:CTGCAGCGCCTTGAAGATG			
*Col2a1*	F:GAGACAGCATGACGCCGAG	62	67	NM_001844.4
	R:GCGGATGCTCTCAATCTGGT			
*Col10a1*	F:CCCTCTTGTTAGTGCCAACC	62	155	[27]
	R:AGATTCCAGTCCTTGGGTCA			
*Runx2*	F:ATACCGAGTGACTTTAGGGATGC	62	131	[27]
	R:AGTGAGGGTGGAGGGAAGAAG			
*β-catenin*	F:AATGCTTGGTTCACCAGTG	62	176	[17]
	R:GGCAGTCTGTCGTAATAGCC			
*GAPDH*	F:TGCCCCCGACCGTCTAC	60	110	[28]
	R:ATGCGGTTCCAGCCTATCTG			

### Western blot analysis

Total protein was extracted from the samples 4 weeks post-induction by using lysis buffer containing 10% sodium dodecyl sulfate (SDS, Affymetrix Inc, Santa Clara, CA, USA), 0.1 M dithiothreitol (DTT, Invitrogen), 1% glycerol, 1.2% urea, and 1 M Tris-HCl pH 7.4 and complete protease inhibitor. The total protein concentration was determined by using the Bradford assay [29]. Twenty micrograms of total protein were separated on 10% SDS-PAGE, followed by electro-transfer to nitrocellulose membrane (BioRad). The membranes were exposed to blocking buffer (5% skim milk in PBS with 0.1%Tween-20 (PBST)) and then incubated with either anti-human type II collagen (dilution 1:1,000, clone 6B3, Chemicon) or anti-human β-actin (dilution 1:1,000, Millipore). Membranes were incubated with (goat anti-rabbit or -mouse) secondary antibody conjugated to alkaline phosphatase (dilution 1:20,000) and were then developed by using 5-Bromo-4-chloro-3-indolyl phosphate/Nitro blue tetrazolium (SIGMA FAST^™^ BCIP/NBT).

### Statistical analysis

All experiments were repeated three times. Statistical analyses were performed using SPSS 17.0 (SPSS, Inc., Chicago, IL, USA). Data are presented as mean ± standard deviation of independent experiments. Statistical difference was analyzed using one-way analysis of variance (ANOVA) with Tukey’s HSD Post Hoc Test. *P-value* <0.05 was considered significant.

## Results

### Isolation and characterization of hWJ-MSCs

**Fig 1:**
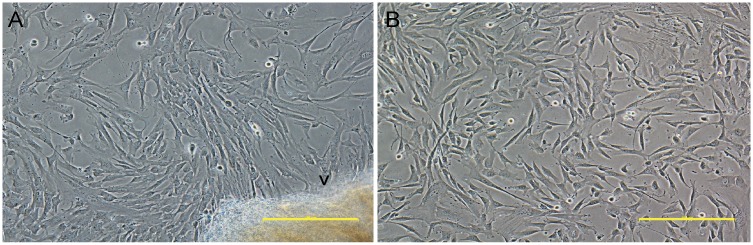
Morphology of hWJ-MSCs with a typical fibroblast-like morphology. **(A)** Phase contrast images of hWJ-MSCs expanded from Wharton’s jelly tissue (arrow) and **(B)** hWJ-MSCs at 80% confluences. Scale bar = 10 μm.

**Fig 2:**
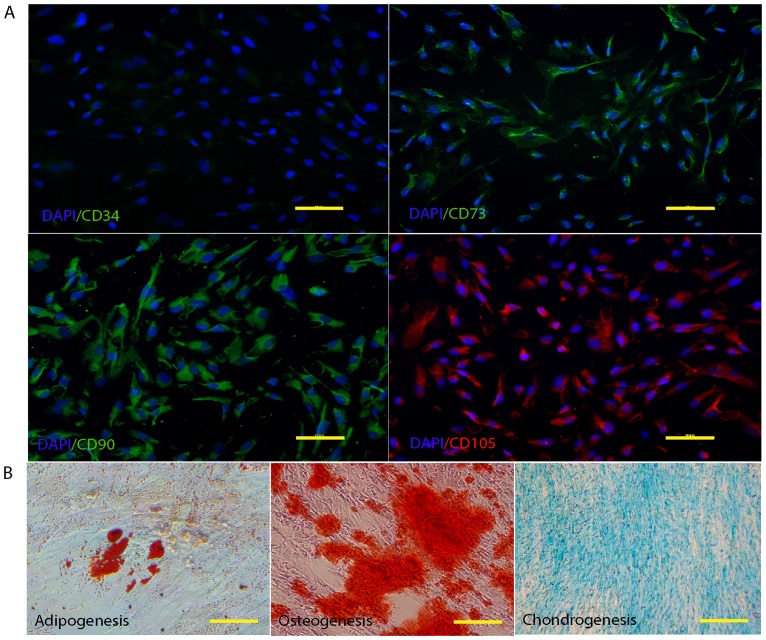
Characterization of hWJ-MSCs. **(A)** Immunophenotype of MSCs, immunofluorescent micrographs staining expression of MSC markers (CD73, 90, and 105), Nuclei were counterstains with DAPI (blue). Cells were negative for hematopoietic marker (CD34). Scale bar = 20 μm. **(B)** Differentiation of hWJ-MSCs to mesodermal linage cells. The cells were induced to undergo adipogenic, osteogenic, and condrogenic differentiation.

MSCs were isolated from WJ tissue of the human umbilical cord. Primary MSCs derived from WJ tissue were adherent, spindle shape, and fibroblast-like cells. The outgrowths of the cells were observed after 5–7 days of culture (Fig 1A). When the cells reached 80% confluence (Fig 1B), they were harvested and expanded for further usage. Based on the MSC properties using standard criteria for the identification of MSCs [28], hWJ-MSCs were positive for the MSC markers, CD73, CD90, and CD105, and negative for the hematopoietic marker, CD34 (Fig 2A). In addition, hWJ-MSCs were induced toward adipocytes, osteoblasts, and chondrocytes to confirm their capacity for MSC differentiation. As shown in Fig 2B, the lipid droplets formation was demonstrated by Oil Red O staining. Calcium deposit was visualized by Alizarin Red. Moreover, the accumulation of GAGs was examined by Alcian blue staining.

### Effect of LiCl and SB216763 on hWJ-MSC viability

**Fig 3:**
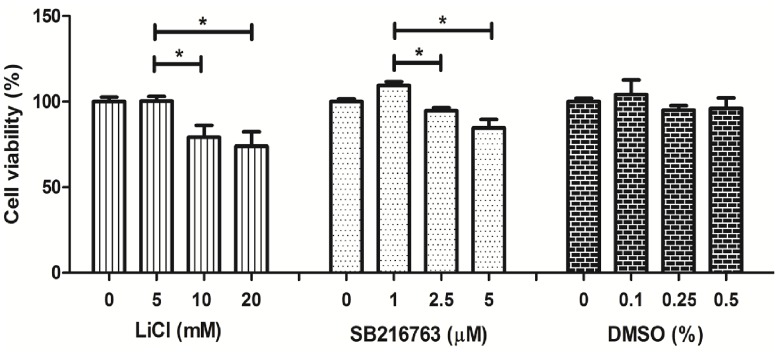
The toxicity effect of LiCl and SB216763 on hWJ-MSC viability. hWJ-MSCs were cultured with 0–20 mM LiCl **(A)**, 0–5 μM SB216763 **(B)**, or 0–0.5% DMSO **(C)**, for 72 hrs in 96-well plate. Then, the viability was detected by MTT assay. **DMSO without chemical was use as vehicle control. **Data were exposed as mean ± SD. *P<0.*05*.

The viability of hWJ-MSCs was determined after culture in the presence of different concentrations of LiCl and SB216763 for 3 days using MTT assay. Culture medium without LiCl and SB216763 was used as control. Data are expressed as percent (%) normalized to the control condition without chemical supplementation. For the vehicle control, the cells were treated with DMSO (0.1–0.5%). The results indicated that DMSO did not decrease hWJ-MSC viability as shown in Fig 3. However, LiCl and SB216763 presented cytotoxic effects. In the presence of LiCl, the viability of hWJ-MSCs was reduced in a dose-dependent manner. In the presence of 5 mM LiCl, cell viability was 100.3 ± 2.73% which was significantly higher than that in the presence of 10 and 20 mM LiCl (79.17 ± 7.00%, 73.96 ± 8.5%, respectively). These results indicated that high LiCl concentrations, 10 and 20 mM, could induce hWJ-MSC death. In the presence of SB216763, the cell viability significantly decreased in a dose-dependent manner. In the presence of 1 μM SB216763, cell viability was 109.38 ± 2.36%, which was significantly higher than that in the presence of 2.5 and 5 μM SB216763 (95.64 ± 1.79% and, 84.52 ± 5.14%, respectively) (Fig 3). Hence, for further experiments, 5 mM of LiCl and 1 μM SB216763 were selected to study the effects of LiCl and SB216763 on chondrogenic differentiation.

### Effect of LiCl and SB216763 on chondrogenic differentiation

**Fig 4:**
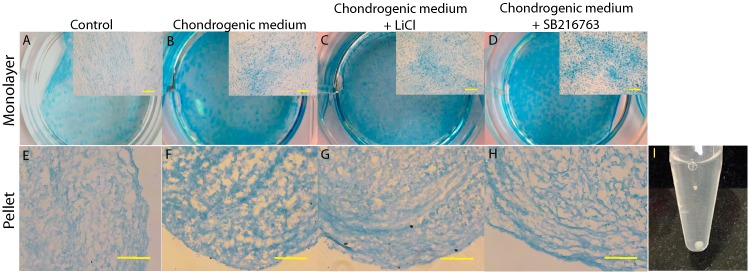
Accumulation of GAGs was stained by alcian blue. **(A-D)** Photographs of monolayer expanded cells cultured for 2 weeks. Scale bar = 10 μm. **(E-H)** Pellets culture at 4 weeks after differentiation. Scale bar = 20 μm. **(I)** Morphology of pellet culture at 4 weeks after differentiation.

Chondrogenic differentiation of hWJ-MSCs was confirmed by Alcian blue staining for GAG matrix synthesis that is an important ECM component of the cartilage tissue. hWJ-MSCs were cultured in different culture media, including chondrogenic medium and chondrogenic medium supplemented with LiCl or SB216763. Monolayer and pellet cultures were grown in the medium. After 2 weeks of monolayer experiment, positive staining with Alcian blue was identified in all treatment groups except for the control group (Fig 4A–4D). Strong staining was observed in chondrogenic medium + SB216763, a weaker blue staining was observed in cells cultured in chondrogenic medium + LiCl and chondrogenic medium alone. For pellet experiments, after 4 weeks, the morphology has a white shiny cartilage-like appearance (Fig 4I). We further stained the pellet sections with Alcian blue (Fig 4E–4H). Alcian blue staining of GAGs was observed in all treatment groups, except for the control group.

**Fig 5:**
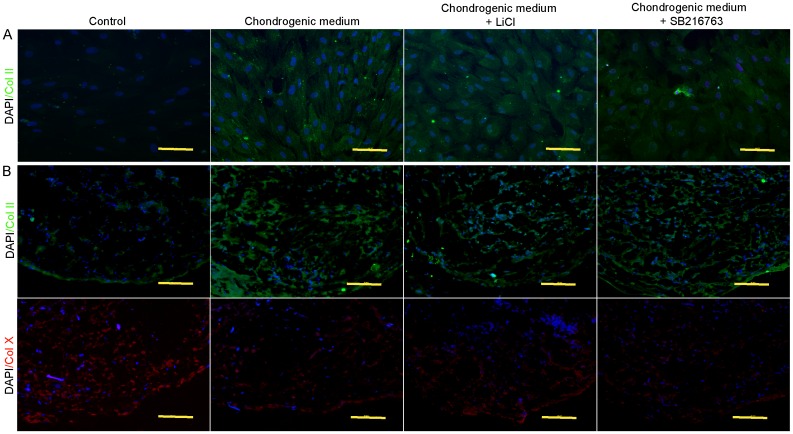
Immunofluorescent staining of cartilage specific type collagen. **(A)** immunofluorescent staining for collagen type II in monolayer expanded cultured on 3 weeks after differentiation. Scale bar = 20 μm. **(B)** Collagen type II and X expressions in pellet experiment on 4 weeks after differentiation. Scale bar = 20 μm.

**Fig 6:**
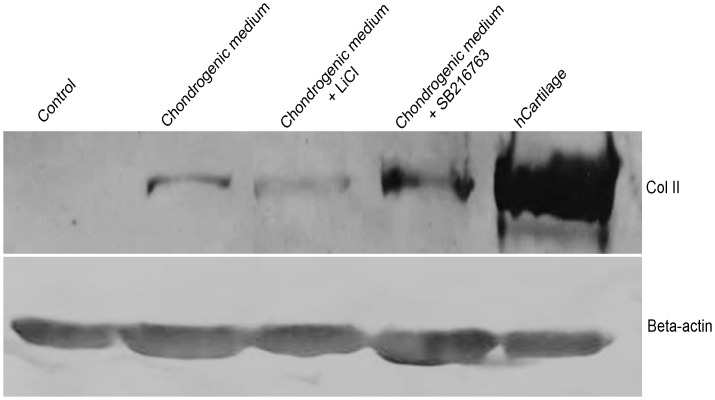
The collagen type II protein after 4 weeks of inductions examined by western blot analysis. β-actin was used as an internal control.

To further analyze chondrogenic differentiation, we performed immunofluorescence staining for a cartilage specific marker, collagen type II (Fig 5). In monolayer experiments, an increase in the expression of collagen type II was detected in the treatment groups, in which cells develop a dense filamentous matrix network. This was not observed in the control group (Fig 5A). We also observed a substantial number of collagen type II positive cells in the pellet experiments (Fig 5B). Interestingly, collagen type II expression clearly increased in the treatment groups. However, in the control group, no tissue-like morphology was observed and cells were negative for collagen type II. Collagen type II expression was confirmed by western blot analysis. As shown in Fig 6, collagen type II expression was stronger in cells cultured in the chondrogenic medium + SB216763 than in cells cultured in the chondrogenic medium + LiCl and chondrogenic medium alone.

**Fig 7:**
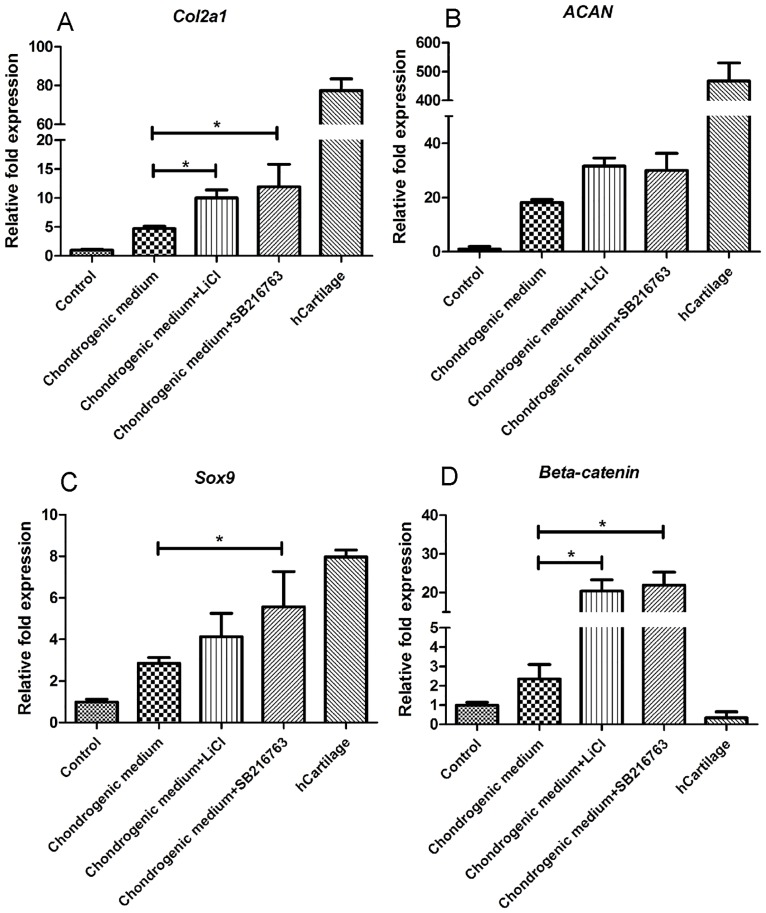
qPCR analysis for chondrogenic gene expressions after 4 weeks of inductions. **(A)** *Col2a1*, **(B)** *ACAN*, **(C)** *Sox9* and **(D)** *β-catenin*. Gene expression was normalized to coresponding *GAPDH* and calculated by relative expression compared to control cells. The experiments were perfromed three times. **Data were expressed as mean±SD, *P<0.*05*.

In addition, the expression of chondrogenic genes (*Col2a1*, *ACAN*, and *Sox9*) was investigated. Gene expression was normalized to *GAPDH* and calculated as the relative expression compared to the control group. *Col2a1* expression significantly increased in cells cultured in the chondrogenic medium + SB216763 (12-fold) and chondrogenic medium + LiCl (10-fold) groups compared to cells cultured in the chondrogenic medium alone (4-fold) (Fig 7A). No significant difference was observed between the chondrogenic medium + SB216763 group and the chondrogenic medium + LiCl group. *ACAN* expression increased in both groups (chondrogenic medium + SB216763 and chondrogenic medium + LiCl) and reached 30-and 31-fold when compared to the control group (Fig 7B), respectively. However, no significant difference was observed when compared to cells cultured in the chondrogenic medium alone (18-fold). In the chondrogenic medium + SB216763 group, *Sox9* expression increased by 5-fold and was significantly different than that in the chondrogenic medium alone group (2-fold), while that of the chondrogenic medium + LiCl group was not significantly different (4-fold) than that of the control group (Fig 7C).

### Effect of LiCl and SB216763 on the Wnt signaling pathway

We next examined the expression of members of the Wnt/β-catenin signaling pathway. *β-catenin* expression increased in the chondrogenic medium + SB216763 and chondrogenic medium + LiCl groups, reaching 22-and 20-fold that of the control group, respectively (Fig 7D). In both groups, β-catenin expression was significantly higher than that of cells cultured in the chondrogenic medium alone (2-fold). These results indicated that LiCl and SB216763 are able to induce the Wnt signaling pathway by increasing β-catenin expression.

### LiCl and SB216763 treatments suppressed the progression of chondrocyte hypertrophy

We also investigated the effects of LiCl and SB216763 on chondrocyte hypertrophy markers. The pellet sections were stained with anti-human collagen type X, a marker of hypertrophic chondrocytes developing from the osteogenic lineage. Cells in the control group developed normally to the osteogenic linage as evidence by strong collagen type X expression. However, in the treatment groups, positive staining was not or only slightly observed in the pellet sections (Fig 5B).

**Fig 8:**
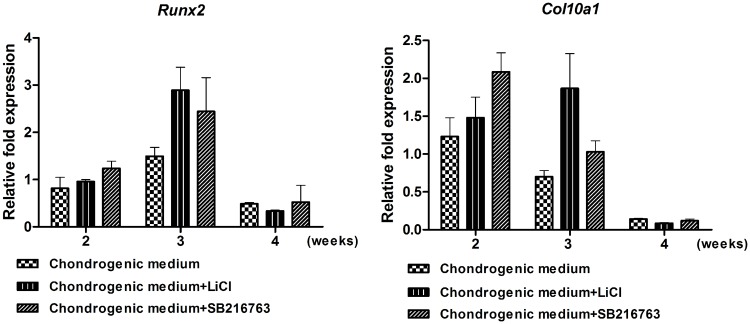
Expression level of hypertrophic marker genes (A) *Col10a1*, and (B) *Runx2* was quantified by qPCR after 2, 3, and 4 weeks of inductions. Gene expression was normalized to coresponding *GAPDH* and calculated by relative expression compared to control cells. Data were expressed as mean±SD, The expperiments were perfromed three times.

In addition, qPCR was used to examine the expression of markers of the development of chondrocytes derived from hWJ-MSCs to the hypertrophic state. We collected samples at 2, 3, and 4 weeks after differentiation to evaluate *Runx2* and *Col10a1* expression. We observed a modest and transient enhancement in *Col10a1* and *Runx2* mRNA levels, which was more evident in chondrogenic medium + SB216763 and chondrogenic medium + LiCl groups after 2 weeks of induction. At later time-points of differentiation (4 weeks of induction), *Col10a1* and *Runx2* expression decreased in all treatment groups (Fig 8A and 8B). These results indicated that LiCl and SB216763 did not induce hypertrophic differentiation of hWJ-MSCs, while promoting chondrogenesis.

## Discussion

Degenerative articular cartilage remains one major problem worldwide, especially for the elder. Regeneration requires chondrocytes to repair the erosion of the ECM and improved cartilage repair. The use of chondrogenically differentiated MSCs has been proposed as a therapeutic strategy for cartilage regeneration [30]. Here, we investigated the influence of LiCl and SB216763 synergistically with TGF-β3 on chondrogenic differentiation of hWJ-MSCs.

The Wnt signaling pathway modulates chondrogenesis and cartilage development [12] by regulating chondrocyte proliferation and differentiation, and by maintaining the cell phenotype [31]. GSK-3 inhibition by LiCl or SB216763 promotes β-catenin accumulation and induces the Wnt signaling pathway [23,24]. β-catenin accumulation activates transcription in conjunction with co-transcription factors, LEFs/TCFs [32]. It was previously reported that LiCl (5–10 mM) can effectively induce the canonical Wnt signaling pathway and mediate cell differentiation of MSCs [16,17,33] and articular chondrocytes [34]. SB216763 has been shown to regulate cell proliferation and survival and to induce the transcription of β-catenin-dependent genes [24].

Several studies reported that the crosstalk between Wnt signaling and other signaling pathways can modulate chondrogenesis. Fischer and colleagues demonstrated that Wnt3A in combination with BMP-2 can enhance chondrogenesis in mMSCs [13]. Narcisi and colleagues also reported that Wnt3A in combination with FGF-2 supported the long-term expansion and enhanced chondrogenic potential in MSCs [18]. Eslaminejad and colleagues reported that combination of TGF-β with LiCl or SB216763 induced chondrogenic differentiation, demonstrated by an increase in the expression of *Sox9*, *ACAN*, and *Col2a1*. Proteoglycan levels were also evaluated during chondrogenic differentiation of MSCs from the bone marrow [17]. Our results showed that treatment of hWJ-MSCs with LiCl or SB216763 synergistically with TGF-β3 to induce chondrogenic differentiation up-regulated the expression of cartilage-specific markers, including *ACAN*, *Col2a1*, and *Sox9* as well as GAG accumulation in the monolayer and pellet experiments. Western blot analysis revealed that the production of collagen type II was increased. However, the results of this study came from only one patient. Samples from different patient donors might have different efficiency, proliferation capacity, and potential for differentiation [35]. Since MSCs from umbilical cords are easy to isolate and has expansion potential. It would be a choice candidate for cell-based therapies in the future. This study reveals the mechanisms by which TGF-β3 affects the Wnt/β-catenin signaling pathway, promoting the chondrogenic differentiation of hWJ-MSCs. TGF-β3 is known as a major inducer promoting chondrogenic differentiation by inducing the downstream phosphorylation of Smad2/3, which directly leads to the induction of chondrogenesis due to the stabilization of the Sox9 transcription complex by Smad2/3 [36,37]. TGF-β can independently or cooperatively regulate LEF/TCF target genes in the Wnt signaling pathway and these pathways can synergistically activate target genes [38]. Another report showed that β-catenin signaling induces transcriptional activity and promotes chondrogenic differentiation in a Sox9-dependent manner [39]. Sox9 plays an important role directly regulating the expression of the cartilage genes, *Col2a1* and *ACAN*, during chondrogenesis [40].

The Wnt/β-catenin signaling pathway plays a crucial role in the progression of chondrocyte hypertrophy. Our results showed that LiCl or SB216763 treatment suppressed the progression of chondrocyte hypertrophy as evidenced by decreased expression of *Col10a1* and *Runx2* markers. These results are in agreement with a study from Yang and colleagues showing that the continuous co-activation of two signaling pathways inhibited chondrocyte hypertrophy by suppressing the expression of *Col10a1*, *Runx2*, and alkaline phosphatase, and did not lead to ossified tissue *in vivo* [16]. However, Kawata and colleagues showed that activation of the Wnt/β-catenin signaling pathway in chondrocytes by Wnt3a or SB216763 inhibits GSK-3 phosphorylation and decreased the expression of *ACAN* and *Col2a1*, while increasing the expression of *Col10a1* and *MMP-13* [19]. In this study, cells were not cultured with any TGF-β supplements. The supplementation of any TGF-β stimulates the early chondrogenic differentiation but they inhibit the terminal differentiation of chondrocytes. Mueller and colleagues reported that TGF-β3 having the highest chondrogenic potential of all isoforms, and their action results in rapid cell differentiation [41].

Prolonged activation of Wnt signaling pathway promotes chondrocyte maturation. This study showed that cultured cells with GSK-3 inhibitors (Wnt agonist) increased the expression of β-catenin to induce the Wnt signaling pathway, while the cartilage chondrocytes from the patient expressed a very low level. It might be not improved homeostasis to repair tissue. In previously study showed that Wnt signaling pathway are involved in supporting repair processes by maintaining a stem cell pool and specifying cell fates in other organs [39,42], it would be a similar function in the tissue repair after transplantation.

Both LiCl and SB216763 act as GSK-3 inhibitors, thereby initiating the Wnt signaling pathway. LiCl is a chemical compound that has already been approved as a therapeutic and used for the treatment of patients with bipolar disorder [43]. Our results showed that the expression of collagen type II was strongly increased in the chondrogenic medium + SB216763 as evidence by western blot analysis. It also up-regulated the expression of several genes, including *ACAN*, *Col2a1*, and *Sox9*. SB216763 was more effective than LiCl treatment, as previously reported [17]. SB216763 is a synthetic small molecule that can rapidly diffuse across cell membranes, reach intracellular sites of action, and specifically target the signaling pathway [44].

In conclusion, GSK-3 inhibitors (LiCl and SB216763) can induce the Wnt signaling pathway and promote chondrogenic differentiation of hWJ-MSCs in the presence of TGF-β3, without inducing chondrocyte hypertrophy. These results indicate that LiCl and SB216763 are choice candidates for further *in vivo* therapeutic trials and would be of great importance for cartilage regeneration.
